# Si-QD Synthesis for Visible Light Emission, Color Conversion, and Optical Switching

**DOI:** 10.3390/ma13163635

**Published:** 2020-08-17

**Authors:** Chih-Hsien Cheng, Gong-Ru Lin

**Affiliations:** 1 Department of Electrical Engineering, Graduate Institute of Photonics and Optoelectronics, National Taiwan University, Taipei 10617, Taiwan; f97941009@ntu.edu.tw; 2 NTU-Tektronix Joint Research Center, National Taiwan University and Tektronix Inc., Taipei 10617, Taiwan

**Keywords:** Si quantum dots (Si-QD), light emitting diode (LED), porous Si, Si ion implantation, plasma enhanced chemical vapor deposition

## Abstract

This paper reviews the developing progress on the synthesis of the silicon quantum dots (Si-QDs) via the different methods including electrochemical porous Si, Si ion implantation, and plasma enhanced chemical vapor deposition (PECVD), and exploring their featured applications for light emitting diode (LED), color-converted phosphors, and waveguide switching devices. The characteristic parameters of Si-QD LED via different syntheses are summarized for discussion. At first, the photoluminescence spectra of Si-QD and accompanied defects are analyzed to distinguish from each other. Next, the synthesis of porous Si and the performances of porous Si LED reported from different previous works are compared in detail. Later on, the Si-QD implantation in silicide (SiX) dielectric films developed to solve the instability of porous Si and their electroluminescent performances are also summarized for realizing the effect of host matrix to increase the emission quantum efficiency. As the Si-ion implantation still generates numerous defects in host matrix owing to physical bombardment, the PECVD method has emerged as the main-stream methodology for synthesizing Si-QD in SiX semiconductor or dielectric layer. This method effectively suppresses the structural matrix imperfection so as to enhance the external quantum efficiency of the Si-QD LED. With mature synthesis technology, Si-QD has been comprehensively utilized not only for visible light emission but also for color conversion and optical switching applications in future academia and industry.

## 1. Introduction

**Figure 1:**
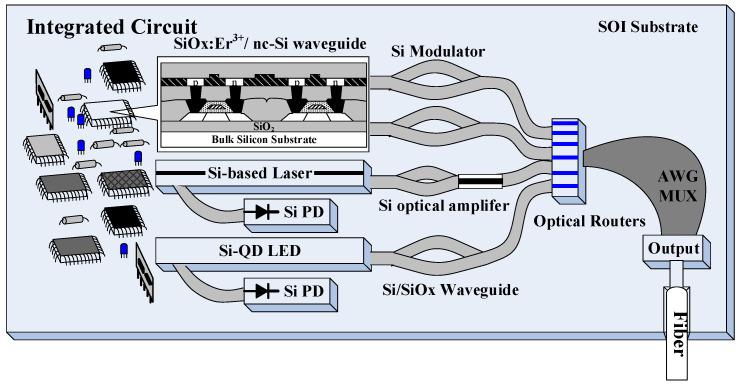
Schematic diagram of all-Si based photonic integrated circuit.

Silicon (Si) is the most popular semiconductor comprehensively to be employed as various electronic and photonic devices. The data transmission rate gradually approaches the upper limitation of the copper wire when the spatial resolution of device pattern continuously increases to reduce chip size. The integrated Si photonics can help to take pace with Moore’s law. Therefore, they can serve as one promising solution to overcome the current bottleneck on the evolution of microelectronic data communication, as shown in Figure 1. However, the crystalline Si (c-Si) can hardly perform active optical functionality including efficient lasing and amplification owing to its indirect bandgap and small exciton binding energy (~10 meV). These reasons lead to weak spontaneous emission with ultralow quantum efficiency at room temperature [1,2]. For the complete integration of photonic and electronic circuits with devices in Si photonics, the quantum-confined emitters become one of the potential research fields in past decades. Specially, the Si nanocrystal (NC) and quantum dot (QD) possess great quantum efficiency. This approach is typically used to release the constrained set by momentum conservation which predominates the indirect-bandgap optical transition. That is because the band diagram broadening occurs via spatial-confinement in low-dimensional Si nanostructures. In principle, the categories of the low-dimensional semiconductor system can be classified as the two-dimensional quantum wells, one-dimensional quantum wires, and zero-dimensional quantum dots to induce specific physic mechanisms. The first mechanism is the small size or quantum confinement effect [3,4]. The boundary condition for the crystalline periodicity can be broken up to redistribute the density of states in energy-momentum space when the material size is decreased around or below the optical wavelength, the de Broglie wavelength, and the coherence length of superconducting state. This phenomenon induces nanostructure with decreasing surface atomic density to change most of the material characteristics. Moreover, the quantum size effect can be induced when the crystal size is further decreased to the Bohr radius of exciton [5,6] as approximately 4.5 nm for Si material [7]. If the NC only contains the limited atoms, the interaction among neighboring atoms is weakened to form the discrete energy state via quantum confinement. In this case, the energy gap between the highest occupied and the lowest unoccupied molecular orbitals (HOMO and LUMO) can be broadened by shrinking the NC size. Simultaneously, the surface effect can be effectively enhanced to induce the higher chemical activity by enlarging the ratio of the surface atoms to total atoms when the NC size is suppressed [8,9]. On the basis of the occurrence of abovementioned effects in Si nanostructure, some material parameters such as acoustics, optics, electricity, magnetics, thermodynamics, and mechanics may change or deviate accordingly. For example, the melting point of 1687 K for bulk Si is lowered to 600 K by decreasing the Si structure to a NC size of 4 nm or less [10].

More important, the optical characteristics of Si NC or QD also deviate from those of bulk material including the broadened absorption spectrum, the blue-shifting bandgap energy, and the enhanced quantum confinement, etc. The Si-NC also increases its unsaturated dangling bonds as it exhibits large specific surface to degrade the average coordination number. This contributes to the broadband distribution on the vibration mode of bonding resonance instead of single and preferable vibration modes. For Group-IV semiconductor NCs or QDs with their broadening absorption spectra by decreasing the NC size [11,12], two mechanisms are responsible for their blue-shifting peaks. One is the sizing effect by shrinking the nanostructure size to enlarge its energy gap, and another is the surface effect with enlarging the surface tension to deform the crystal lattice. The bonding length of the Si NC or QD can be shortened to increase its vibration frequency via reducing the lattice constant for blue-shifting the whole absorption band [13]. The mean free path of electrons is limited because of the quantum confinement by shrinking the radius of Si NC or QD below the Bohr radius. The electrons easily combine with holes to form exciton via the induced overlap of electron-hole wave functions when they are confined in such small region. In principle, the overlapping coefficient is increased to shorten the recombination time (*τ_R_*) for increasing the oscillator strength (*f_osc_*(*ω*)) of exciton in Si NC or QD, as described by [14]:

(1) $$f_{osc}(\omega )=\frac{2\pi \varepsilon_{0}mc^{3}}{e^{2}n\omega^{2}}\cdot \frac{1}{\tau_{R}},$$

where *ε_0_* denotes the dielectric constant in vacuum, c the light speed in vacuum, e the electron charge, *n* the refractive index of Si, *ω* the angular frequency, and *m* the exciton mass equivalent to the summation of the effective masses for electron (*m_e_*) and hole (*m_h_*) in the weak quantum confinement regime, where *m_e_* and *m_h_* are respectively, 0.19 *m_0_* and 0.286 *m_0_*, with *m_0_* denoting the free electron mass [15,16]. Decreasing the Si NC or QD size enlarges the oscillator strength of exciton to enhance the absorption coefficient in the exciton band. In addition, the recombination mechanisms are also affected by the quantum confinement effect [17] as the localized recombination predominates the carrier behavior prior to their diffusion into defects. This effect suppresses the Shockley–Read–Hall recombination. In addition, the Auger recombination can hardly happen unless two excitons concurrently exist in the same Si NC or QD. These phenomena urge the fast radiative recombination process for the efficient visible light emission from group-IV semiconductors NC or QD [18,19,20]. Therefore, the new era of investigation has been developed toward their related devices with strong photo- and electro-luminescence (EL) [21,22,23,24,25,26,27,28,29,30,31]. The content of this review paper is divided into five categories for discussion. In Section 2, the luminescence mechanisms of Si-QDs are comprehensively overviewed. Then, the performances of the light emitting diode (LED) made by nano-porous Si are discussed in Section 3. In contrast, the optical and electrical properties of LEDs made by Si-ion-implanted dielectric films with buried Si-QDs are presented in Section 4. At last, the discussion on lighting performances of the Si-QD in various silicide semiconductor or dielectric layer via plasma-enhanced chemical vapor deposition are given in Section 5.

## 2. Photoluminescence of Si-QD

In general, the photoluminescence (PL) of Si-QD is mainly dominated by the quantum confinement effect to induce the direct band-to-band transition. However, the minor effect is sometimes caused by luminescent structural defects such as the weak oxygen bond, the neutral oxygen vacancy, the precursor of Si-QDs and the non-bridging oxygen hole center under different syntheses. For the quantum confinement effect, the varied displacement of energy via changing the QD size effectively scales down the valence band level and moves up the conduction band level to enhance the bandgap energy. At early stage of development, the porous Si was the first candidate for generating the efficient PL because of its low dimensionality with survived Si skeleton. Among the impressive researches on the efficient PL from porous Si [6,32,33,34,35,36,37,38,39,40], Canham demonstrated the most distinguished work to use electrochemical and chemical dissolution methods for the mesoporous Si layer and the Si quantum wire array fabrication as early as 1990 [6]. Under the excitation at 514.5 nm for 6 h, the Si quantum wire array preserves its PL at 762 nm (1.62 eV) [6]. In 1991, Bsiesy et al. also demonstrated the PL of porous Si at 560 nm, and further used the electrochemical oxidation process to stabilize the luminescent characteristics [32]. Moreover, Tsai et al. observed that the PL of porous Si was measured between 750 and 800 nm after annealing at different temperatures [33]. Koshida et al. detuned the chemical etching parameters to anodize the porous Si for shifting its PL spectra to the shorter wavelength via the stronger quantum size effect [34]. In 1996, Mizuno further employed the postanodization illumination without adding any oxidation process for 15 min to obtain blue PL emission to 400 nm [35].

**Figure 2:**
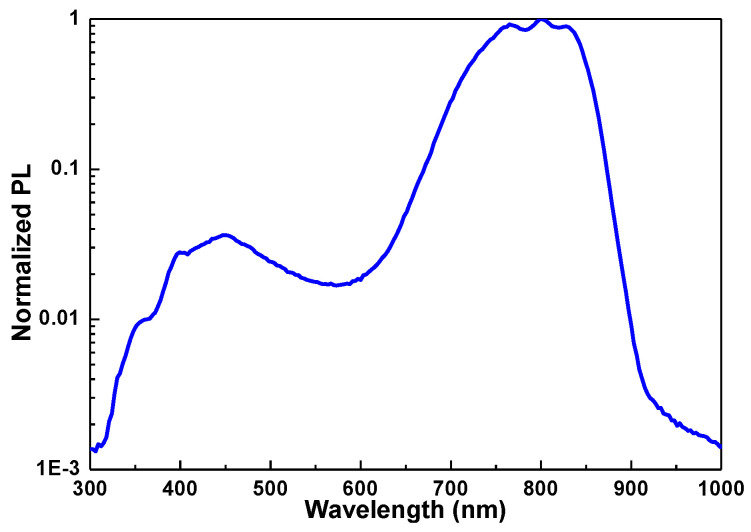
The photoluminescence (PL) spectrum of silicon quantum dots (Si-QDs) in Si-implanted SiO_2_ host matrix.

In addition to the porous Si structure, the buried Si-QDs in the host matrix have also been observed to provide efficient visible and near-infrared (IR) luminescence as the host matrix with larger bandgap confines the Si-QDs with the smaller bandgap. This phenomenon was known to induce the stronger quantum confinement for Si-QDs. In 1995, Mutti et al. used the Si^+^-ion implantation and post-annealing to synthesize the buried Si-QDs in SiO_2_ matrix. Then, they increased the annealing temperature to red-shift the PL peak from 490 to 640 nm [41]. In 1996, Min et al. adjusted the dose concentration from 2 × 10^16^ to 5 × 10^16^ cm^−2^ to enrich the Si-QDs in the SiO_2_ film [42]. The PL of the buried Si-QD in SiO_2_ matrix was varied from 650 to 790 nm after deuterium passivation and annealing at 1100 °C [42]. Shimizu-Iwayama et al. also confirmed that the PL peak at 729 nm (1.7 eV) was contributed by the Si-QDs in Si-implanted SiO_2_ film [43]. In 1999, Linnors et al. reported the radiative recombination time of 10–150 μs for the 689–775 nm (1.6–1.8 eV) PL from Si-QDs in Si-implanted SiO_2_ [44]. Lin et al. discriminated the PL from defects (410–460 nm and 520 nm) and Si-QD (820–850 nm) in Si-implanted SiO_2_ [45]. For example, the PL spectroscopy of Si-QDs in Si-implanted SiO_2_ film is also observed in Figure 2. It indicates that the PL peak can be blue-shifted to 725 nm for the buried Si-QDs in Si-implanted SiO_2_ film after annealing at 1100 °C in 3 hr. In addition, the defect-related PL peak is also observed at 450 nm.

**Figure 3:**
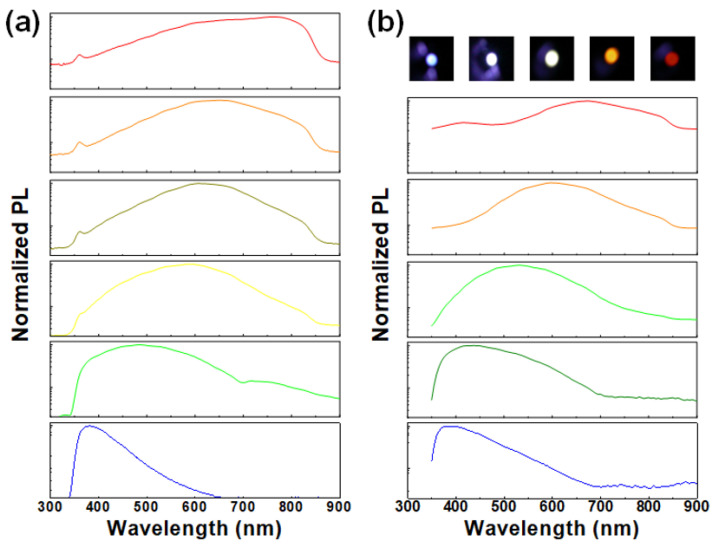
Normalized PL spectra of the buried Si-QDs in (**a**) SiO_x_ and (**b**) SiN_x_ host matrix.

In addition to Si-ion implantation, other methods including sputtering [46,47,48,49,50], e-beam evaporation [51,52], and plasma-enhanced chemical vapor deposition (PECVD) [53,54,55,56,57,58,59,60,61,62] are employed to grow the Si-QDs in host matrix. In 2004, Wu et al. utilized the radio frequency (RF) magnetron sputtering to synthesize Si-QDs in SiO_2_ matrix with 2–4 nm size for 533–716 nm PL after post-annealing at 600 °C [46]. In particular, Samanta et al. fabricated the SiO_x_ nanowire with buried Si-QDs by using the DC sputtering. The observed PL peak can be blue-shifted from 669 to 575 nm owing to the spatially confinement of Si-QDs within SiO_x_ nanowire [47]. In 2009, Hao et al. further grew the Si-QD/SiO_2_ multilayer film via the RF sputtering for sandwiching the Si-QDs with SiO_2_ matrix to provide the red PL emission with a peak wavelength of 800 nm [48]. Moreover, the microcrystalline Si was synthesized in SiO_x_ film via the e-beam evaporation and post-annealing in N_2_ and O_2_ gaseous mixture [51]. Similar process was performed by using the thermal evaporation for amorphous SiO_x_ film deposition and post annealing at 1000 °C for Si-QD formation. The buried Si-QDs with an average size of 4.3 nm were formed to generate the broadband red PL emission between 754 and 882 nm [52]. In comparison with sputtering and evaporation, the PECVD method can perform the precise synthesis for the composition ratio variation by adjusting various parameters such as substrate temperature, reactant fluence, RF plasma power, etc. Such a parametric tool facilitates to flexibly grow the buried Si-QDs with precisely controlled sizes in any host matrix. For example, Iacona et al. varied the N_2_O/SiH_4_ fluence ratio in PECVD from 6 to 10 to synthesize the SiO_x_ film with different composition ratio [53]. With 1100 °C annealing for Si-QD precipitation, the PL peak is observed to blue-shift from 900 to 650 nm [53]. Alternative approach was proposed by changing the thickness of Si layer during the deposition of Si/SiO_2_ superlattice. The Si-QD size can be enlarged from 0.9 nm to 2.3 nm for red-shifting the PL peak from 800 to 910 nm [54]. Similarly, the thickness of SiO_x_N_y_ layer in SiO_x_N_y_/SiO_2_ film can be adjusted for controlling the Si-QD size from 2.5 to 7 nm and red-shifting the PL peak from 729 to 886 nm [55]. Later on, the N_2_O/SiH_4_ fluence ratio was varied from 6.8 to 2.3 during the SiO_x_ growth to control its composition ratio. This can effectively reduce Si-QD size in SiO_x_ film with enlarging the O/Si composition ratio such that the less Si content can hardly generate the larger Si-QD in SiO_x_ film [56]. This method helps to enhance the quantum confinement for improving the blue-shift of PL spectrum. With increasing the O/Si composition ratio, a huge PL blue-shift of the buried Si-QD is obtained from 760 to 380 nm [56]. On the contrary, the RF plasma power during the PECVD growth for the Si-QD in SiO_x_ film can be enlarged from 20 W to 70 W to shrink the Si-QD size from 4.5 to 1.7 nm [57]. In our work, Figure 3a exhibits that the PL peak of the buried Si-QDs can be detuned from 800 to 380 nm by adjusting the fabricating parameters during growth.

In contrast to SiO_x_, either SiN_x_ or SiC_x_ is also considered as host matrix to confine the Si-QDs [63,64,65,66,67,68,69,70,71,72,73,74,75,76]. In 1992, Chen et al. utilized the PECVD and Ar^+^-laser annealing to fabricate the Si:H/SiN_x_:H multiple quantum well (MQW). This MQW structure effectively suppresses a full width at half-maximum (FWHM) of PL to only 5 nm [63]. Another approach varied the flow rates of SiH_4_ and N_2_ gas to detune the Si-QD size [64]. Reducing the Si-QD size from 6.1 to 2.7 nm significantly blue-shifts the visible PL peak from 850 to 410 nm [64]. Negro et al. used the PECVD method and post-annealed at 700 °C for 60 min to form the Si-QDs in Si-rich SiN_x_ with corresponding PL at 850 nm [65]. In our work, the flexible control on N/Si composition ratio and Si-QD size is demonstrated via the adjustment of NH_3_ fluence during PECVD synthesis. With increasing the NH_3_ fluence from 170 to 250 sccm to vary the N/Si composition ratio from 0.85 to 1.32, the observed PL peaks can be blue-shifted from 675 to 385 nm, as shown in Figure 3b. By using the SiC matrix and changing the Si-QD layer thickness in Si-QD/a-SiC superlattice, the Si-QD can shrink its size from 10 to 3 nm for emitting the PL from 1170 to 775 nm [70]. Coscia et al. synthesized the buried Si-QDs in a-SiC via changing the RF plasma power to generate the 636–827 nm PL [71]. Huang et al. changed the SiH_4_ fluence from 0.5 to 8 sccm to suppress the C/Si composition ratio and increase the Si-QD size for blue-to-green shifting PL wavelength from 440 to 520 nm [72]. Tai et al. further demonstrated the Si-rich SiC synthesis for obtaining red PL between 630 and 660 nm from Si-QDs and 480 nm for SiC-QDs [73,74].

Instead of the Si-QD related PL, the radiative-defect related weak PL by the contribution of structural or interfacial defects in SiO_x_ or SiN_x_ host matrices is comprehensively investigated to realize their bonding mechanisms. Particularly, these defect-related PL emissions are usually located between green- and blue-light regions. The PL spectra of the weak oxygen bond (WOB, denoted as O-O) and neutral oxygen vacancy (NOV, denoted as O_3_≡Si-Si≡O_3_) are ranged between 415 and 455 nm [77,78,79,80,81,82]. As a supporting evidence, Cheang-Wong et al. obtained the visible PL peak at 415 nm in Ir^2+^-doped silica glass owing to the contribution of the WOB defect [77]. Lin et al. also observed the PL at the same wavelength from Si^+^-implanted SiO_x_ film [78]. In addition, similar PL peak was also observed in SiO_x_/SiN_x_ superlattice [79]. Tohmon et al. claimed that the PL of NOV defects in oxygen-deficient high-purity silica glass at 459 nm [80]. The blue PL for NOV defects not only in the Si-implanted SiO_2_ film [81] but also in the RF sputtered Si-rich SiO_x_ film was observed at 460 nm [82]. Other than WOB and NOV defects, the precursor of Si-QDs (E’_δ_, denoted as Si↑Si-Si) and the non-bridging oxygen hole center (HBOHC, denoted as O_3_≡Si-O●) defects also contribute to the green and yellow PL [83,84,85,86]. The PL of E’_δ_ defect in the Si-implanted SiO_x_ film without annealing was observed at 653 nm by Shimizu-Iwayama et al. in 1994 [83]. Sakurai et al. analyzed another green PL of the E’_δ_ in oxygen-deficient silica glass at 554 nm [84]. On the other hand, Skuja measured the 653-nm PL of HBOHC defects in silica glass under different temperature condition [85]. For confirmation, Song et al. also reported the PL around 630 nm for the HBOHC defects in Si-implanted SiO_x_ film [86]. In addition to the SiO_x_ host matrix, there are some other defects with radiative recombination in SiN_x_ host matrix [87,88,89]. For example, the PL of Si dangling bonds (denoted as K^0^ center) is located in the middle of bandgap [87]. In addition, the N_4_^+^ center is located close to the conduction band and the N_2_^0^ center state is close to the valence band [88]. Notably, the PL emission of these K^0^, N_4_^+^, and N_2_^0^ centers is dependent on the varied optical bandgap of SiN_x_ host matrix by detuning the composition ratio of SiN_x_ film [89]. However, most of their luminescence can be vanished after the annealing recrystallization.

## 3. Porous Si LED

**Figure 4:**
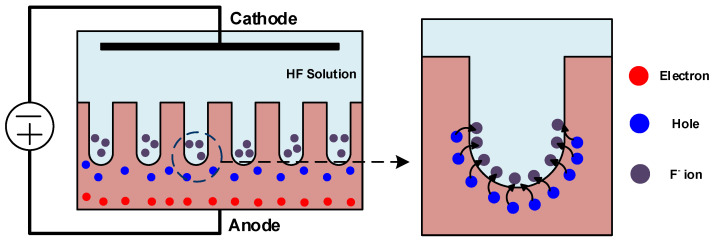
Experimental setup of the anodic electrochemical etching method to form the porous Si.

In early years, the fabrication of the Si-QD LED mainly relies on the development status of the porous Si substrate. With using the anodic electrochemical etching process shown in Figure 4, the hydrofluoric acid (HF) at the anode reacts with the crystalline Si to form the porous Si. The porous or island Si structure is generated upon the surface and the Si pillar structure is formed at the flank during the anodic electrochemical etching. Such an etching procedure etches down the Si wafer along direction of its crystalline axis without causing the variation on other directions. However, the doped Si film such as the p-type Si with more holes can somewhat affect the etching process. Gardelis et al. used the anodic electrochemical etching to fabricate the p-type porous Si with a corresponding PL of 709 nm [90]. Dimova-Malinovska et al. found the short-wavelength PL peak of same p-type porous Si at 690 nm [91]. Because of the n-type Si with more electrons, the etching without assistance of illumination or bias can increase more holes to accelerate the etching process. In 1990, Lehmann and Föll utilized tungsten lamping for the enhanced etching formation of porous Si [92]. Theunissen also confirmed that the n-type Si with >10^18^ cm^−3^ donor concentration can suffer from the anodic dissolution for hole generation below 10-V bias operation [93]. On the other hand, the porous-like Si nanostructure can also be obtained via a metal-assisted chemical etching method alternatively. Under the interaction with single or double phonons, such Si nanostructure reveals a redshifted and broadened Raman scattering peak when comparing with bulk Si, which is in good agreement with the prediction of phonon confinement effect within Si nanostructure [94].

Moreover, the porous Si diameter can be tunable to vary its PL peaks from 770 to 730 nm by changing the HF concentration [95]. With the tungsten lamp illumination to enhance the reaction rate, the porous Si diameter is gradually shrunk to blue-shift its PL from 709 to 310 nm [35]. A similar etching method was utilized to demonstrate the porous Si with its PL at 479 nm, internal quantum efficiency of 0.1%, and a PL decay time of 1 ns [39]. Prokes et al. increased the etching time to 4 h to decrease the porous Si diameter. This phenomenon also contributes to its PL blue-shifting to 697 nm [38]. With the electrochemical etching with a current density of 50 mA/cm^2^ for 10 min, the 10-nm QDs can be observed in porous Si with a pore diameter of 3.5 μm and a pore depth of 60 μm to generate the PL at 750 nm [96]. Such simple fabrication for the porous Si urges a rapid development of the porous Si LED in 1990s. The first porous Si LED grown on n-type Si wafer by using anode electrochemical etching with halogen lamp illumination can emit visible luminescence at 650 nm under biasing at 5 mA and 200 V [97]. By lengthening the anode oxidation time from 1 to 7 s, the porous Si LED with a film thickness of 1–2 μm blue-shifted its EL wavelength blue-shifted from 830 to 700 nm [98]. With the fabrication of indium-tin-oxide (ITO)/porous Si/p-Si/aluminum (Al) LED, the EL at 680 nm with external quantum efficiency (EQE) below 10^−5^% was observed under bias current density of 370 mA/cm^2^ [99]. Apparently, Canham et al. reached a significant progress on the efficient porous Si LED with its EL peak from 740 to 670 nm via increasing the bias from −1.01 to −1.23 V for the maximal EL density of up to 0.1 W/cm^2^ [100]. More important, the respectively maximal PL and EL power conversion efficiencies of 1% and 0.1% were ranked top record then [100]. Later on, Koshida and co-workers changed the contact of porous Si LED to the electropolymerized contact for improving the EL intensity. The porous Si LED exhibited its EL peak at 593 nm with the intensity 2.6 times larger than that using Au contact [101]. Steiner et al. were the first group employing the porous p–n junction structure to enhance the EQE of porous Si LED [102]. With the mesoporous p^+^-Si/nanoporous n-Si/macroporous n-Si structure, the EQE can be effectively improved to 0.01% [102]. In addition, the green and blue EL of porous Si LED at 560 nm and 480 nm was also demonstrated in the same time [103]. Subsequently, Li et al. utilized the conducting polymer contact to replace the Au or ITO contact for improving the EL intensity because it provides relatively higher transparency at emission wavelength of porous Si (630 nm) than Au or ITO [104]. The enhancing EQE of 0.16% from the p^+^–n–n^+^-junction-structured porous Si LED with a corresponding EL peak at 630 nm under a pulsed bias of 20 V was reported by Linnors and co-workers in 1995 [105]. In the same year, Loni et al. also demonstrated the visible EL with its EQE of 0.1% under continuous-wave (CW) operation at 2.3 V and 0.01 A/m^2^ [106]. In 1998, Nishimura et al. further constructed the porous Si LED with a p^+^-n structure to enhance its EQE to 0.8% [107]. With using the semitransparent Au as electrode, the EQE of device can be expected to exceed 1% [107]

In view of previous reports, the porous Si can be regarded as the active layer, however, the porous Si is easily oxidized to degrade its EL stability as the extremely large porous Si surface reacts with abundant oxygen molecules [108,109,110,111,112]. In 1996, Tsybeskov et al. passivated the porous Si surface by annealing the layer at 800–900 °C to extend the LED stability without suffering from power degradation during pulsed operation over one month [108]. Another method employed thin transparent alumina layer to protect the porous Si surface for achieving the CW operation over one month [109]. Alternative approach via the anodic oxidation to oxidize the porous Si surface has also emerged to effectively enhance the EQE to 0.21% and extend its operation time to 8 min with an intensity decay of <10% [110]. A similar approach employed the electrochemically oxidizing thin porous Si layer to enhance the EQE over 1% with intensity degradation by only 8% within 120 s [111]. Other works on the surface passivation of porous Si layer were demonstrated via the covalent bonding with organic monolayers. This method maintains the unchanged EL intensity of porous Si LED during 2-h operation [112]. In 2006, the high-pressure water vapor annealing technology was also developed to maintain the EL intensity without decay during 20-min operation [113]. Even though tremendous efforts were paid to improve the device stability, its EQE is still too low to enable its practical application. 

## 4. Si-Implanted Si-QD LEDs

**Figure 5:**
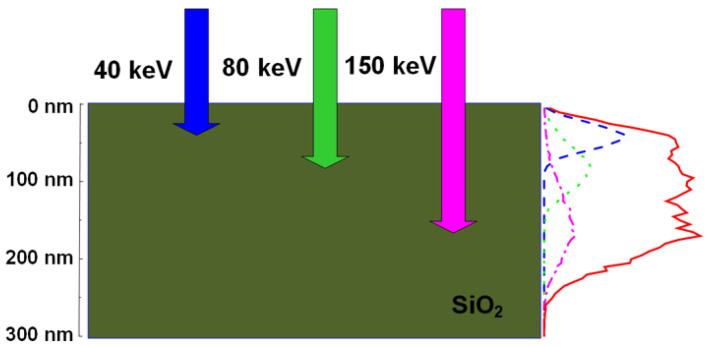
Schematic diagram for the multiple Si ion-implantation with different dose concentrations and implantation energies into SiO_2_ layer to form the Si-QDs.

To take over porous Si LED with instable and low EQE during long-term operation, the Si-ion implantation into SiO_2_ or Si_3_N_4_ host matrix for the Si-QD formation has emerged in the beginning of 21st century. This method self-aggregates these excessive Si^+^ ions as Si-QDs with their sizes tuning by changing the dose concentration of Si^+^ ions. At beginning, Shimizu-Iwayama and co-workers varied the dose concentration from 1 × 10^17^ to 4 × 10^17^ ions/cm^2^ for Si-implanted SiO_x_ synthesis. With increasing dose concentration of Si^+^ ions to enlarge the Si-QD size in SiO_x_ film, the red-shifted luminescent variation was observed under room-temperature operation [83]. Unfortunately, the implanted Si^+^ ions only distribute in a very shallow layer beneath the surface of host matrix. For example, the Si^+^-ions with dose concentration of 5 × 10^16^ ions/cm^2^ and energy of 40 keV can only cover a depth of 60 nm from the SiO_2_ surface with non-uniform distribution of implantation ions according to the transportation of ions in matter (TRIM) software simulation [114]. Such shallow and non-uniform distribution can be overcome through multiple ion-implantation with different dose concentrations and energies. This method can approach the depth of 350 nm with employing different implantation energies at same Si dose concentration of 10^16^ ions/cm^2^ [115]. By taking one example in detail, the implanted dose recipes of Si^+^ ions were set as 5 × 10^15^ ions/cm^2^ at 40 keV, 1 × 10^16^ ions/cm^2^ at 80 keV, and 2.5 × 10^16^ ions/cm^2^ at 150 keV to uniformly distribute the excessive Si atoms in SiO_x_ layer with its depth between 10 and 200 nm from the surface [116]. As shown in Figure 5, using the lower (higher) dose concentration and smaller (larger) implantation energy makes the Si^+^ ions stay with shallower (deeper) depth from SiO_2_ surface with narrower (broader) distribution. With post-annealing the Si-implanted SiO_x_ for aggregating the Si-QDs, recrystallizing the host matrix and suppressing the structural defects in host matrix, numerous studies of the Si-implanted Si-QD LED have been reported [117,118,119,120,121,122,123,124,125,126,127].

**Figure 6:**
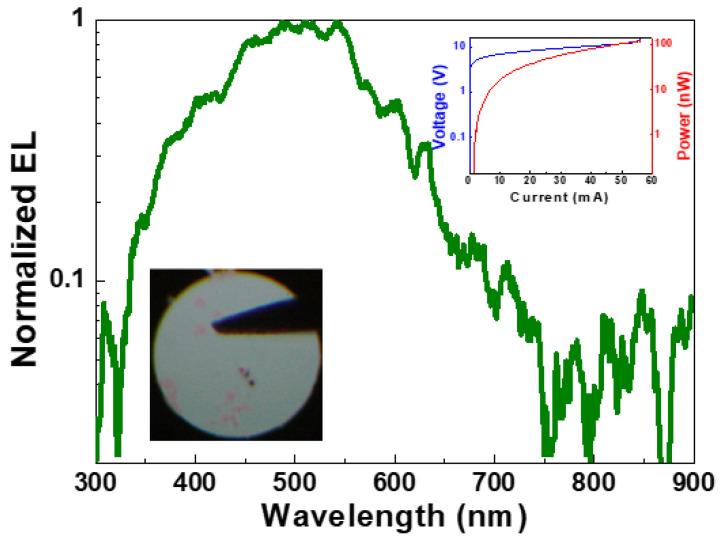
Electroluminescence (EL) spectrum of Si-implanted Si-QD light emitting diode (LED) with its corresponding EL pattern, voltage-current and power-current curves (Inset).

In 1996, the Si-implanted Si-QD in SiO_2_ layer with a thickness of 300 nm was synthesized by using a recipe with Si^+^-ion dose concentration of 2 × 10^16^ ions/cm^2^ and implantation energy of 120 keV to perform EL at 620 nm under a bias at 15 V [117]. A latter experiment used 200-keV Si^+^-ions with dose concentration of 3 × 10^16^ ions/cm^2^ as first recipe and 100-keV Si^+^-ions with 1.8 × 10^16^ ions/cm^2^ as the second recipe to increase the excessive Si content in SiO_2_ film. Then, the fabricated Si-QD LED performed its EL at 455 nm under a bias with current of 100 nA and voltage of 370 V [118]. In contrast, Song and co-workers utilized Si^+^-ions implantation at 25 keV with dose concentration of 1 × 10^16^ ions/cm^2^ and post-annealed the Si-implanted SiO_2_ film with temperature increasing from 100 °C to 1100 °C [119]. The LED exhibited the enhancement on the Si-QD-related EL peak at 730 nm and the degradation on the defect-related EL peaks at 470 and 600 nm. With raising the Si^+^ dose concentration from 3 × 10^16^ to 3 × 10^17^ ions/cm^2^ under constant implantation energy of 150 keV to enlarge the Si-implanted SiO_2_ thicknesses from 12 to 18 nm, the Si-implanted Si-QD LED red-shifts its EL peak from 752 to 855 nm under a bias voltage of 16 V, and shortens its EL decay time from 100 to 6 μs [120]. In 2005, Walters and co-workers employed the Si-implanted Si-QDs in SiO_x_ layer as the active layer to implement the metal-oxide-semiconductor LED (MOSLED). This device contains the Si-QDs with average size of 2–4 nm to demonstrate EL emission at 750 nm with a decay time of 2.5 μs under a bias voltage of 6 V [121]. In addition, our work employs the multiple ion-implantation and post annealing to demonstrate the Si-QD dependent EL from Si-implanted SiO_x_ LED. Figure 6 reveals the EL between 400 and 600 nm with the blue emission by the WOB and NOV defects (405–455 nm), green emission via the precursor of Si-QDs (520 nm), and red emission from the larger Si-QDs (600 nm), as shown in Figure 6. The turn-on current and voltage are respectively measured as only 0.2 mA and 5 V, as shown in the inset of Figure 6. The maximal power achieves 100 nW under biasing at 10 V and 70 mA.

Besides, the Si_3_N_4_ host matrix is also employed to fabricate the Si-implanted Si-QDs in SiN_x_ film [124,125,126,127]. In 2009, Cen et al. utilized the multiple ion-implantation with the Si^+^ dose concentrations of 4 × 10^16^ ions/cm^2^ at 25 keV at the first time, 8 × 10^15^ ions/cm^2^ at 8 keV at the second time, and 3 × 10^15^ ions/cm^2^ at 2 keV at the third time into Si_3_N_4_ film to form the Si-implanted Si-QD in SiN_x_ film with an average excess Si concentration of 1.2 × 10^22^ cm^−3^ [124]. This LED exhibits three main EL peaks at 886 nm, 564 nm, and 413 nm [124]. The EL peak at 886 nm is attributed to the Si-QDs and other two EL peaks at 564 and 413 nm are contributed by the structural defects [124]. With the Si^+^ dose concentration enlarging from 2 × 10^16^ ions/cm^2^ to 4 × 10^16^ ions/cm^2^, the excessive Si content in SiN_x_ film also increases to form more Si-QDs. This Si-implanted Si-QD LED exhibits its green-yellow EL and the promoting EQE of 10^−4^–10^−3^% [125,126,127]. Nevertheless, most of the previous reports for the Si-implanted Si-QD LEDs seldom mentioned their EQE as the uniform size distribution of Si-QDs in host matrix is still hardly achieved for providing efficient visible luminescence. In addition, the structural damage in SiO_x_ or SiN_x_ film is too serious such that a lot of irradiative defects appear to scatter or absorb electron-hole pairs for efficient recombination. Therefore, the PECVD-grown Si-QD LED with less damaged structure than the implanted device gradually develops as the main-stream device nowadays.

## 5. PECVD Grown Si-QD LED

**Figure 7:**
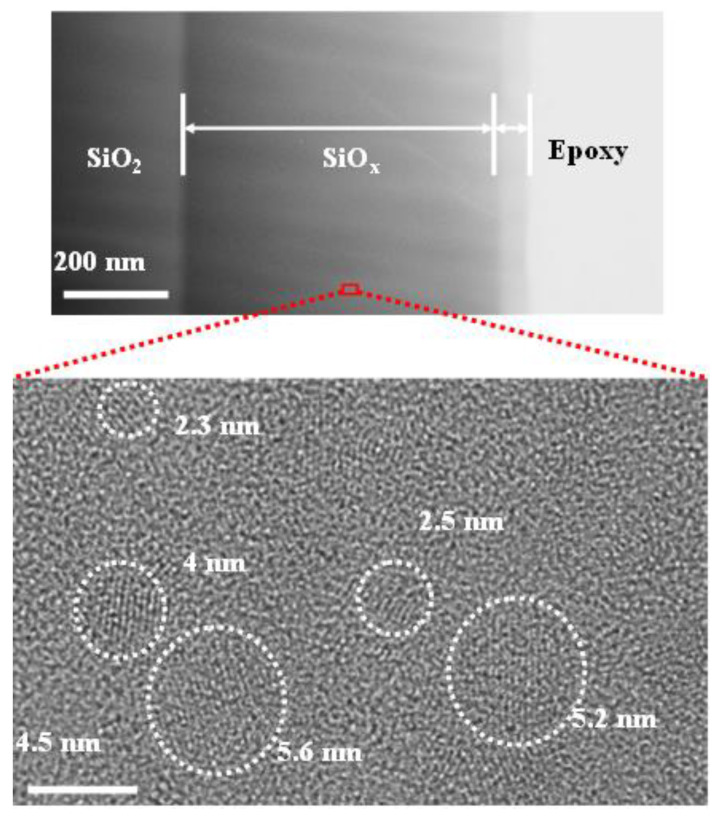
The transmission electron microscopy (TEM) (Upper) and high-resolution TEM (HR-TEM) (Lower) images of plasma enhanced chemical vapor deposition (PECVD)-grown Si-QDs in Si-rich SiO_x_ film.

In the 2000s, the PECVD-grown Si-QD LED has emerged not only because of its reduced structural imperfection but also because of its precise control on the Si-QD size distribution. In addition, the PECVD-grown Si-QDs can more uniformly exist in host matrix as compared to other syntheses. For example, the transmission electron microscopy (TEM) image of the PECVD-grown Si-QDs in Si-rich SiO_x_ film is shown in the upper part of Figure 7. From the upper part of Figure 7, the thickness of Si-rich SiO_x_ layer can be evaluated as 200 nm. To observe the Si-QDs in Si-rich SiO_x_, the high-resolution TEM (HR-TEM) image of the Si-QDs in Si-rich SiO_x_ film is shown in the lower part of Figure 7. The Si-QDs with their size between 2.4 and 5.6 nm are observed and the Si-QD volume density is estimated as 1.1 × 10^18^ cm^−3^. Versatile orientations of Si-QDs in Si-rich SiO_x_ film are evaluated as (110), (111), (002), and (021) with the corresponding plane spaces of 0.38, 0.31, 0.27, and 0.24 nm, respectively. In comparison with porous Si and implanted Si-QDs, the density and size of PECVD-grown Si-QDs can easily be controlled by varying the reactant gas content, the substrate temperature, and the RF plasma power during growth. As a result, the PECVD becomes the most common technology for the Si-QD synthesis [128,129,130,131,132,133,134,135,136,137,138,139,140,141,142,143]. In 2002, Franzò et al. employed SiH_4_ and N_2_O molecules as reactant gaseous for the growth of the Si-rich SiO_x_ sample, and performed the post-annealing at 1100–1250 °C to form the Si-QDs [130]. For the Si concentration of 46% in Si-rich SiO_x_ film, the EL peak of SiO_x_ MOSLED with buried 1-nm Si-QDs can be observed at 850 nm under biasing at 50 V and 0.2 A/cm^2^ [130]. In addition, the SiO_x_ MOSLED with average Si-QD size of 4 nm exhibited its EL peaks at 700 nm and maximal output power of 48 nW with a corresponding P–I slope of 0.84 mW/A under a bias at 86 V [130]. The EQE can be also obtained as 1.6 × 10^−3^% [131]. The Si nanopyramids were also used at SiO_x_/Si interface for decreasing the turn-on voltage and increasing the turn-on current density to enhance the current injection and recombination efficiency. This method significantly enhanced its EL power to 30 nW and maintained the EL intensity over 10 h [132,133]. In 2006, Perálvarez et al. fabricated the Si-QDs with an average size of 3.6 nm and a volume density of 5 × 10^17^ cm^−3^ in Si-rich SiO_x_ film as luminescent centers to achieve its EL peak at 816 nm, EL decay lifetime of 5 μs, and EQE of 0.03% [134].

**Figure 8:**
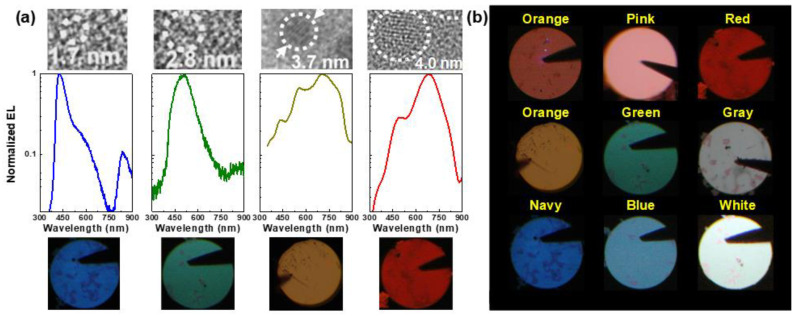
(**a**) EL spectra of Si-QD metal-oxide-semiconductor LEDs (MOSLEDs) with their corresponding EL patterns and Si-QD size. (**b**) Versatile EL emission patterns of Si-QD MOSLEDs.

Similar material was employed to demonstrate the Si-QD MOSLED with two main EL wavelengths of 740 and 1000 nm [135]. In 2007, Barreto et al. also constructed the 815-nm Si-QD MOSLED with its turn-on voltage of 15 V and EQE of 0.03% [136]. Lin et al. also utilized the CO_2_ laser annealing to generate the Si-QDs in SiO_x_ MOSLED [137]. Three main EL peaks at 590, 715, and 810 nm are contributed by different-size Si-QDs to achieve the maximal output of 50 nW under a bias at 85 V and 2.3 mA/cm^2^ [137]. The Si nanopillars with a size of 30 nm, a height of 350 nm, and an area density of 2.8 × 10^10^ cm^−2^ were employed in Si-QD MOSLED to enhance the current injection. This device obtained its maximal output power of 700 nW with a corresponding P–I slope of 2.8 ± 0.8 mW/A to achieve the EQE of 0.1% under biasing at 0.375 mA [138]. In 2008, Chen and co-workers employed the Si-QD/SiO_2_ multilayers to enhance the EL intensity of the Si-QD p-i-n LED by 50 times than single-layered Si-QD LED [139]. In 2009, Anopchenko et al. further used the SiO_2_/Si-QD in SiO_x_ multilayers to demonstrate the power efficiency of 0.01% for Si-QD MOSLED under 1-μA bias operation [140]. With increasing the bias voltage from 2.5 to 6 V, the EL peak can be blue-shifted from 916 to 827 nm [140]. Moreover, the EL peak of MOSLED can be blue-shifted from 700 to 430 nm by suppressing the average Si-QD size from 4 to 1.7 nm to achieve the maximal output power of 1 μW and the EQE of 2.4% [141,142,143]. In our work, decreasing the Si-QD size can effectively blue-shift the EL peak with a corresponding EL pattern from red to blue, as shown in Figure 8a. Therefore, adjusting the fabricating parameters to detune the Si-QD size demonstrates versatile-color Si-QD MOSLED, as shown in Figure 8b.

In recent years, the SiN_x_ dielectric material becomes another host matrix to demonstrate the Si-QD LED although the SiN_x_ material has the lower energy bandgap to degrade the quantum confinement effect [144,145,146,147,148,149,150,151,152]. In 2001, Park and co-workers fabricated the Si-QDs in SiN_x_ to exhibit its EL peak at 620 nm and turn-on voltage of <5 V, and the EQE of 2 × 10^−3^% [144]. In 2005, Cho et al. used the SiH_4_ and NH_3_ molecules as reactant gaseous to fabricate the Si-QDs in Si-rich SiN_x_ film. The Si-QD LED obtained its EL at 600 nm, maximal output power of 2.3 mW, and maximal EQE to 1.6% under biasing at 70 mA [145]. The periodic micron-scale rugged SiN_x_ patterns were fabricated on the surface of Si-QD LED to increase the light extraction efficiency by 2.8 time than the flat-surfaced Si-QD LED [146]. In addition, the Ni/Au contact was also used to enhance the carrier injection for improving EQE by 10−65% as compared to the amorphous Si-QD LED [147]. Huh et al. further utilized the 2.5-nm-thick Ag interlayer inserting between the indium tin oxide (ITO) contact layer and SiC doping layer to enlarge the output power by 40% [148]. Moreover, the undoped SiC layer was added between the Si-QD active layer and n-type SiC layer to enhance the output power [149]. In 2010, Lin et al. compared the lighting performance of SiN_x_ and SiO_x_ LED [150]. Owing to the lower barriers at Si-QD/SiN_x_ and Al/SiN_x_ interfaces, the SiN_x_ LED has a lower turn-on voltage of 10.45 V to easily escape the electrons and holes from Si-QD to decrease the EQE [150,151].

**Figure 9:**
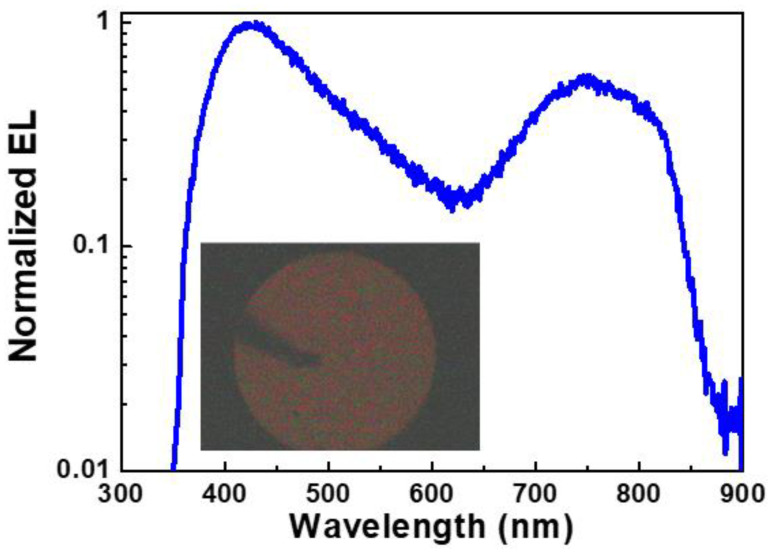
EL spectra of Si LEDs by using the SiN_x_ film as host matrix. Inset: The photographs of corresponding EL emission pattern.

**Figure 10:**
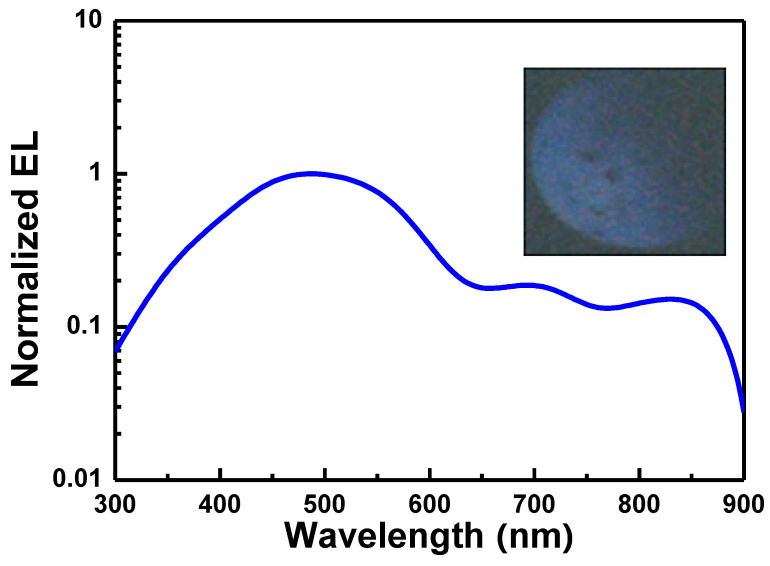
EL spectrum of Si LED by using the SiC film as host matrix. Inset: The photographs of corresponding EL emission patterns.

In our work, the Si-QD LED exhibits its EL peak at 740 nm because of the contribution of Si-QDs, as shown in Figure 9. In addition, the EL peak at 420 nm is attributed to the structural defects in SiN_x_ host matrix. In 2012, Huang et al. also fabricated the Si-QDs with an average size of 2.4 nm and an area density of 4.6 × 10^12^ cm^−2^ in Si-rich SiN_x_ film to demonstrate the 710-nm Si-QD LED [152]. Owing to the dielectric host matrix with the relatively large resistivity to decrease the current injection efficiency, the SiC semiconductor was selected as a candidate of host matrices [153,154,155,156,157]. In 2011, Rui et al. detuned the Si-QD from 4.2 to 1.4 nm in SiC host matrix by detuning the C/Si composition ratio and annealing temperature to blue-shift the EL peak of the Si-QD LED from 775 to 539 nm. Moreover, the 2.7-nm Si-QDs in Si-QD/SiC multilayer were used as active layer. This Si-QD LED exhibited its EL peak at 650 nm and improved EL power by 8.6 times than the device with the Si-QD/SiC single layer [154]. Wang and co-workers also fabricated the SiC LED with a p-i-n structure to observe two EL peaks at 689 and 775 nm owing to the contribution of different-size Si-QDs [155]. Cheng et al. changed the substrate temperature from 300 °C to 650 °C during the growth to detune the average Si-QD size from 2.5 to 2.7 nm. This method respectively decreased the turn-on voltage and current of yellow-light Si-QD LED to 4.2 V and 0.42 mA to enhance the maximal output power density to 8.52 μW/cm^2^ with a corresponding P–I curve of 0.75 μW/A [156]. Tai and co-workers further suppressed the thickness of SiC with buried Si-QDs to 50 nm to enhance the EQE to 0.158% [157]. In our work, Figure 10 exhibits the EL peaks at 480, 700, and 850 nm for Si-QD LED owing to the contribution of the different-size Si-QD. From abovementioned works, the performance of Si-QD LED can be improved.

Up until now, the Si-QD LEDs via versatile syntheses are still developed and studied for visible light emission but also for color conversion and optical switching applications in the future. On the basis of abovementioned works, the studies of Si-QD LED approaches more piratical application in recent three years. In 2018, Hsu et al. inserted Si-QDs into Al_2_O_3_ membrane to confine the size distribution. The confined size distribution can suppress the luminescent linewidth [158]. In addition, Zhao et al. utilized the Al_2_O_3_ material as interlayer to suppress the exciton quenching and hole accumulation and reduce the carrier leakage [159]. This interlayer effectively improved the optical density and EQE of Si-QD LED to 14 μW/cm^2^ and 0.1%. In 2018, Ghosh et al. firstly demonstrated the flexible red Si-QD LED with its luminance of 5000 cd/m^2^ and EQE of 3.1% on polyethylene terephthalate substrate [160]. In 2019, the same group used ITO/ZnO/Si-QD/WO_3_/Al multilayer to construct the red Si-QD LED with its EQE of 0.25% and luminance of 1400 cd/m^2^ [161]. This device without any encapsulation can maintain its EL performance under 80% humidity in ambient air over 45 days [161]. In 2020, Zhang further demonstrated the white-light Si-QD LED to provide a possibility to replace the commercial LED in the future [162]. This device exhibited its luminance of 225.8 cd/m^2^ and EQE of 1% under biasing at 2.9 V [162].

## 6. Conclusions

In view of previous progress on the Si-QD based electronics and photonics, various syntheses have been developed to enable versatile applications of the Si-QDs for light emission, color conversion and switching. For efficient visible light emission, the EL emission wavelength of Si-QD LEDs has been demonstrated to be widely tunable from 400 to 1000 nm with corresponding EQE varied from 0.1–2% obtained by optimizing the selected material synthesis and device design for implementing visible and near-infrared LEDs. In addition, the real EL power of Si-QD LEDs was observed between 30 nW and 2.3 mW. However, different syntheses such as porous Si etching, Si-ion implantation, and excessive Si deposition also reveal their inherent limitations and individually contribute to some weakness for developing the Si-QD LED with sufficient EQE and power. Up until now, versatile studies specially on improving the conductive host matrix, transparent contact electrode, and spatially-confined synthesis are still going to enhance the stability and efficiency of the Si-QD LED. For different market demands, the fabricated Si-QD by mature syntheses has been comprehensively utilized not only for visible light emission but also for color conversion and optical switching applications in future academic and industrial applications.
